# Crystal structure and Hirshfeld surface analysis of supra­molecular aggregate of 2,2,6,6-tetra­methyl­piperidin-1-ium bromide with 1,2,3,4-tetra­fluoro-5,6-di­iodo­benzene

**DOI:** 10.1107/S2056989024011502

**Published:** 2025-01-01

**Authors:** Atash V. Gurbanov, Tuncer Hökelek, Gunay Z. Mammadova, Khudayar I. Hasanov, Tahir A. Javadzade, Alebel N. Belay

**Affiliations:** a Excellence Center, Baku State University, Z. Xalilov Str. 23, AZ 1148 Baku, Azerbaijan; b Scientific Research Center, Baku Engineering University, Hasan Aliyev Str. 120, Baku, Absheron AZ0101, Azerbaijan; c Centro de Quimica Estrutural, Instituto Superior Tecnico, Universidade de Lisboa, Av. Rovisco Pais, 1049-001 Lisbon, Portugal; d Hacettepe University, Department of Physics, 06800 Beytepe-Ankara, Türkiye; e Department of Chemistry, Baku State University, Z. Khalilov Str. 23, Az 1148 Baku, Azerbaijan; f Western Caspian University, Istiglaliyyat Str. 31, AZ 1001 Baku, Azerbaijan; g Azerbaijan Medical University, Scientific Research Centre (SRC), A. Kasumzade Str. 14, AZ 1022 Baku, Azerbaijan; h Department of Chemistry and Chemical Engineering, Khazar University, Mahzati Str. 41, AZ 1096 Baku, Azerbaijan; i Department of Chemistry, Bahir Dar University, PO Box 79, Bahir Dar, Ethiopia; Universität Greifswald, Germany

**Keywords:** crystal structure, non-covalent inter­actions, halogen bond

## Abstract

The asymmetric unit of the title compound, C_9_H_20_N^+^·Br^−^·C_6_F_4_I_2_, contains one 2,2,6,6 tetra­methyl­piperidine-1-ium cation, one 1,2,3,4-tetra­fluoro-5,6-di­iodo­benzene mol­ecule, and one uncoordinated bromide anion. In the crystal, the bromide anions link the 2,2,6,6-tetra­methyl­piperidine mol­ecules by inter­molecular C—H⋯Br and N—H⋯Br hydrogen bonds, leading to dimers, with the coplanar 1,2,3,4-tetra­fluoro-5,6-di­iodo­benzene mol­ecules filling the space between them. There is a π–π interaction between the almost parallel benzene rings [dihedral angle = 10.5 (2)°] with a centroid-to-centroid distance of 3.838 (3) Å and slippage of 1.468 Å. No C—H⋯π(ring) inter­actions are observed. A Hirshfeld surface analysis indicates that the most important contributions for the crystal packing are from H⋯F/F⋯H (23.8%), H⋯H (22.6%), H⋯Br/Br⋯H (17.3%) and H⋯I/I⋯H (13.8%) inter­actions. Hydrogen bonding and van der Waals inter­actions are the dominant inter­actions in the crystal packing.

## Abstract 2

The asymmetric unit of the title compound contains one 2,2,6,6 tetra­methyl­piperidine-1-ium cation, one 1,2,3,4-tetra­fluoro-5,6-di­iodo­benzene mol­ecule, and one uncoordinated bromide anion. In the crystal, the bromide anions link the 2,2,6,6-tetra­methyl­piperidine mol­ecules by inter­molecular C—H⋯Br and N—H⋯Br hydrogen bonds, leading to dimers, with the coplanar 1,2,3,4-tetra­fluoro-5, 6-di­iodo­benzene mol­ecules filling the space between them.

## 1. Chemical context

**Figure 1:**
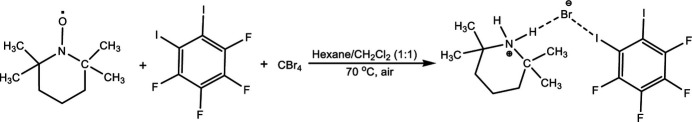
Synthesis of 2,2,6,6-tetra­methyl­piperidin-1-ium bromide from 1,2,3,4-tetra­fluoro-5,6-di­iodo­benzene.

The halogen bond (HaB) is defined as a non-covalent inter­action between the electron-density-deficient region (so-called σ or π hole) of a covalently bonded halogen atom and a nucleophilic (Nu) site in the same (intra­molecular) or another (inter­molecular) mol­ecular entity: *R*—*Ha*⋯Nu [*Ha* = F, Cl, Br or I; *R* = C, *Pn* (pnictogen), *Ch* (chalcogen), metal *etc*.; Nu = lone pair possessing *Ha*, *Ch*, *Pn* or metal atom, π-system, anion, radical, *etc*.; Cavallo *et al.*, 2016 ▸]. Similarly to hydrogen and chalcogen bonds (Gurbanov *et al.*, 2020 ▸; Mahmudov & Pombeiro, 2016 ▸), halogen bonds can also be classified into normal halogen bonds, positive charge-assisted halogen bonds, negative charge-assisted halogen bonds and charge-assisted halogen bonds (Peuronen *et al.*, 2023 ▸). Both the strength and directionality of charge-assisted halogen bonds are much larger than those of normal halogen bonds (Gomila & Frontera, 2020 ▸; Shixaliyev *et al.*, 2014 ▸), which are traditionally regarded as favourable synthetic tools for building new supra­molecular systems (Mahmoudi *et al.*, 2017*a* ▸,*b* ▸). In addition to their catalytic functions (Ma *et al.*, 2021 ▸), *N*-oxide radicals can act as halogen-bond acceptors (Pang *et al.*, 2013 ▸). In the context of this work, we investigated a new negative charge-assisted halogen-bonded supra­molecular aggregate, which was obtained by the reaction of 2,2,6,6-tetra­methyl­piperidinyl-1-oxy (TEMPO) with 1,2,3,4-tetra­fluoro-5,6- di­iodo­benzene in the presence of CBr_4_ in a mixture of hexa­ne/CH_2_Cl_2_ at 343 K (see Fig. 1 ▸). We provide herein a detailed description of the synthesis and an examination of the mol­ecular and crystal structures together with a Hirshfeld surface analysis of the title compound, (I).
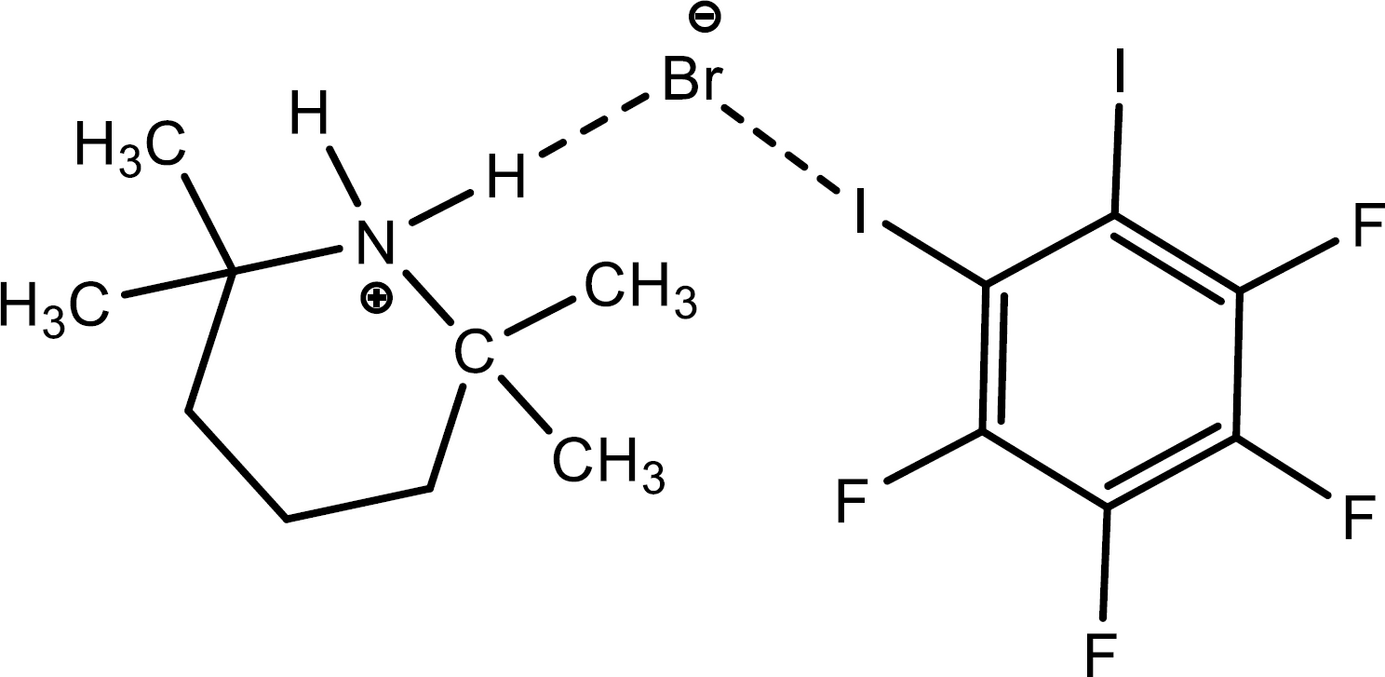


## 2. Structural commentary

**Figure 2:**
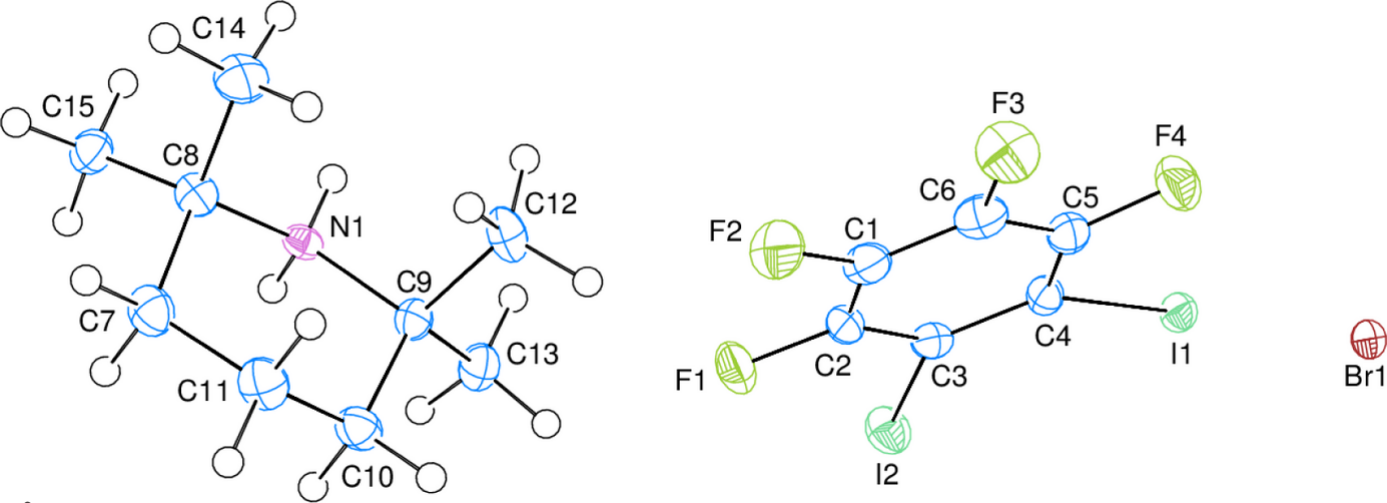
The title compound with atom-numbering scheme and 50% probability ellipsoids.

Two mol­ecules are present in the asymmetric unit of the title compound, 2,2,6,6-tetra­methyl piperidine-1-ium and 1,2,3,4-tetra­fluoro-5,6-di­iodo­benzene, in addition to one uncoordin­ated bromide ion (Fig. 2 ▸). Atoms I1, I2, F1, F2, F3 and F4 are −0.0116 (3), −0.0287 (3), 0.005 (3), −0.022 (3), −0.003 (3) and 0.033 (3) Å, respectively, away from the best least-squares plane of the benzene ring (C1–C6). All atoms of the benzene derivative are essentially coplanar. The piperidine ring (N1/C7–C11), is in a chair conformation. There are no apparent unusual bond distances or inter­bond angles within the two mol­ecules.

## 3. Supra­molecular features

**Figure 3:**
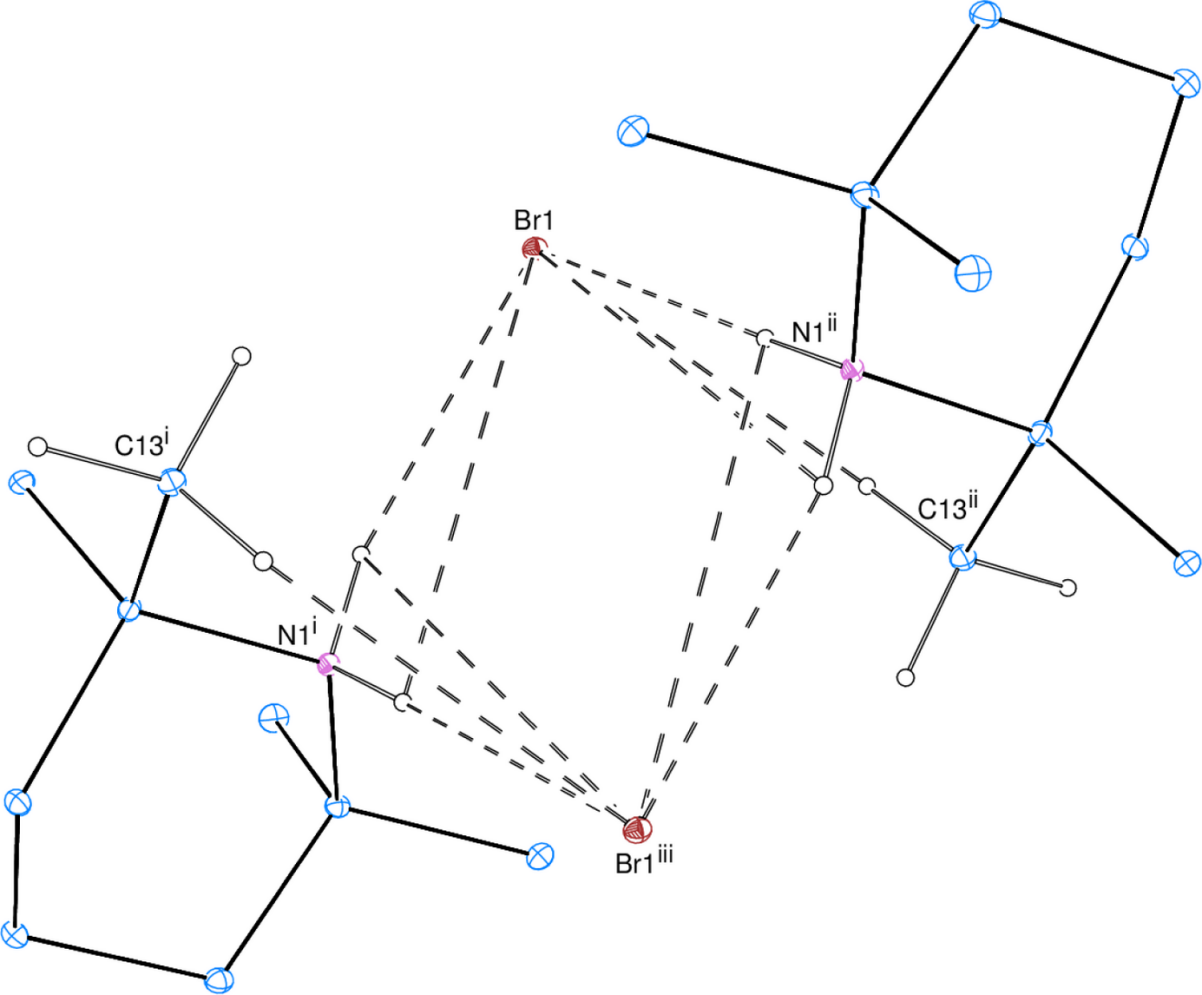
The H⋯Br contacts leading to dimerization. Inter­molecular C—H⋯Br and N—H⋯Br hydrogen bonds are shown as dashed lines. H atoms not involved in these inter­actions are omitted for clarity.

**Figure 4:**
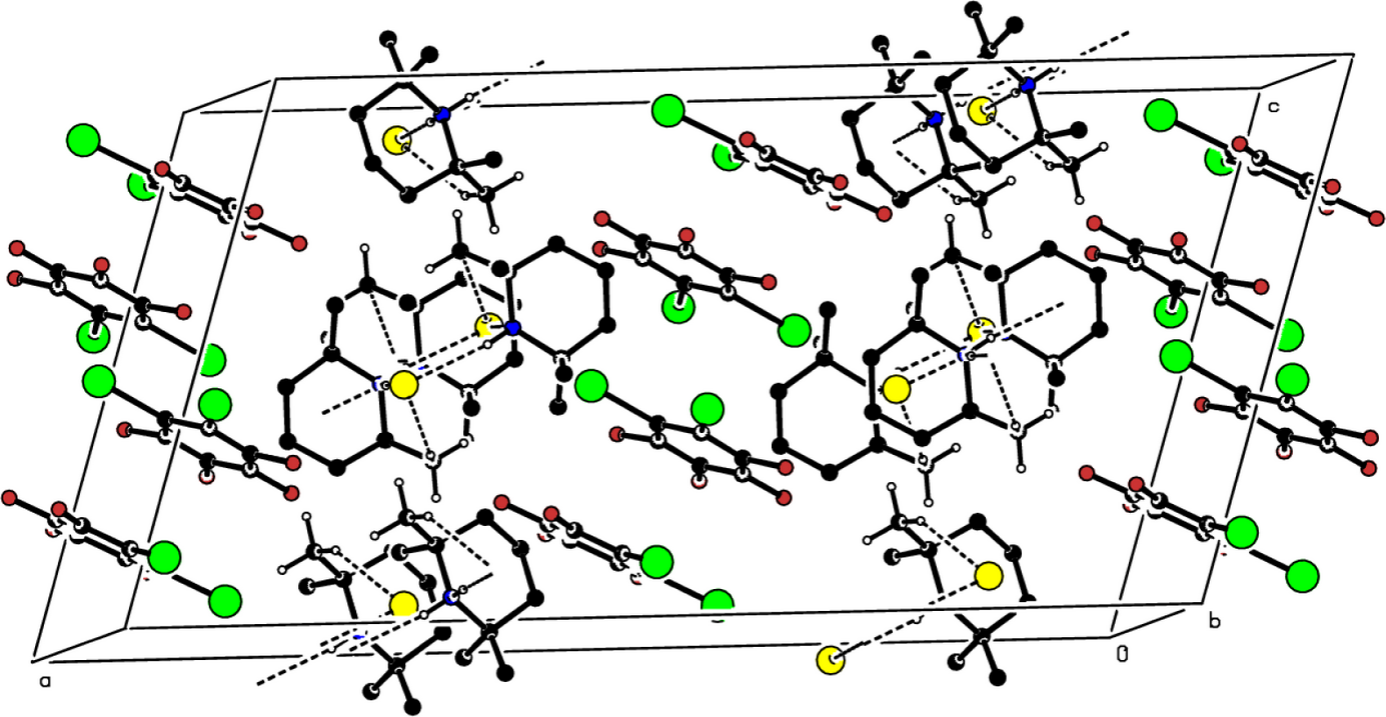
A partial packing diagram, viewed down the *b*-axis direction. Inter­molecular C—H⋯Br and N—H⋯Br hydrogen bonds are shown as dashed lines. H atoms not involved in these inter­actions are omitted for clarity.

With regard to inter­molecular contacts, the uncoordinated bromide ions link the 2,2,6,6-tetra­methyl­piperidine mol­ecules through inter­molecular C—H⋯Br and N—H⋯Br hydrogen bonds (Table 1 ▸) with a double or triple acceptor atom, resulting in dimers (Fig. 3 ▸). In the crystal, the dimers are stacked along the *b*-axis direction, while the coplanar 1,2,3,4-tetra­fluoro-5,6-di­iodo­benzene mol­ecules protrude along the *c*-axis direction, filling the space between the dimers (Fig. 4 ▸). There is a π–π interaction between the C1–C6 benzene rings with a centroid-to-centroid distance of 3.838 (3) Å, where the dihedral angle between the benzene rings is 10.5 (2)° with a slippage of 1.468 Å.

## 4. Hirshfeld surface analysis

**Figure 5:**
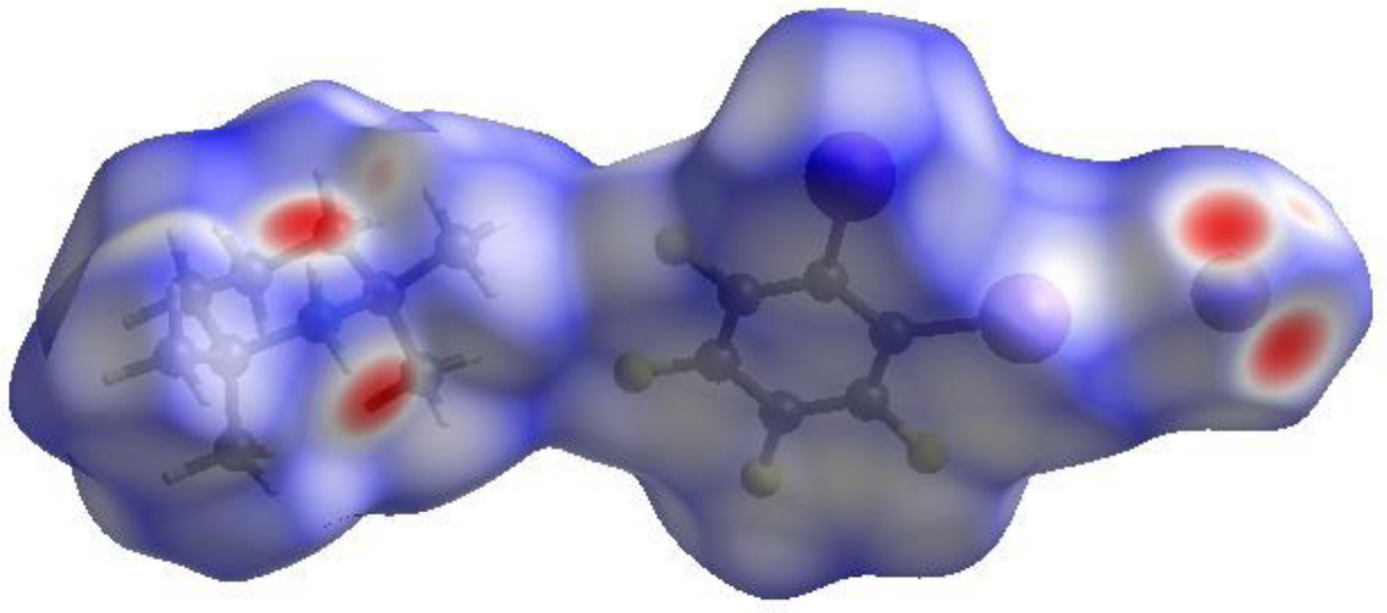
View of the three-dimensional Hirshfeld surface of the title compound plotted over *d*_norm_.

**Figure 6:**
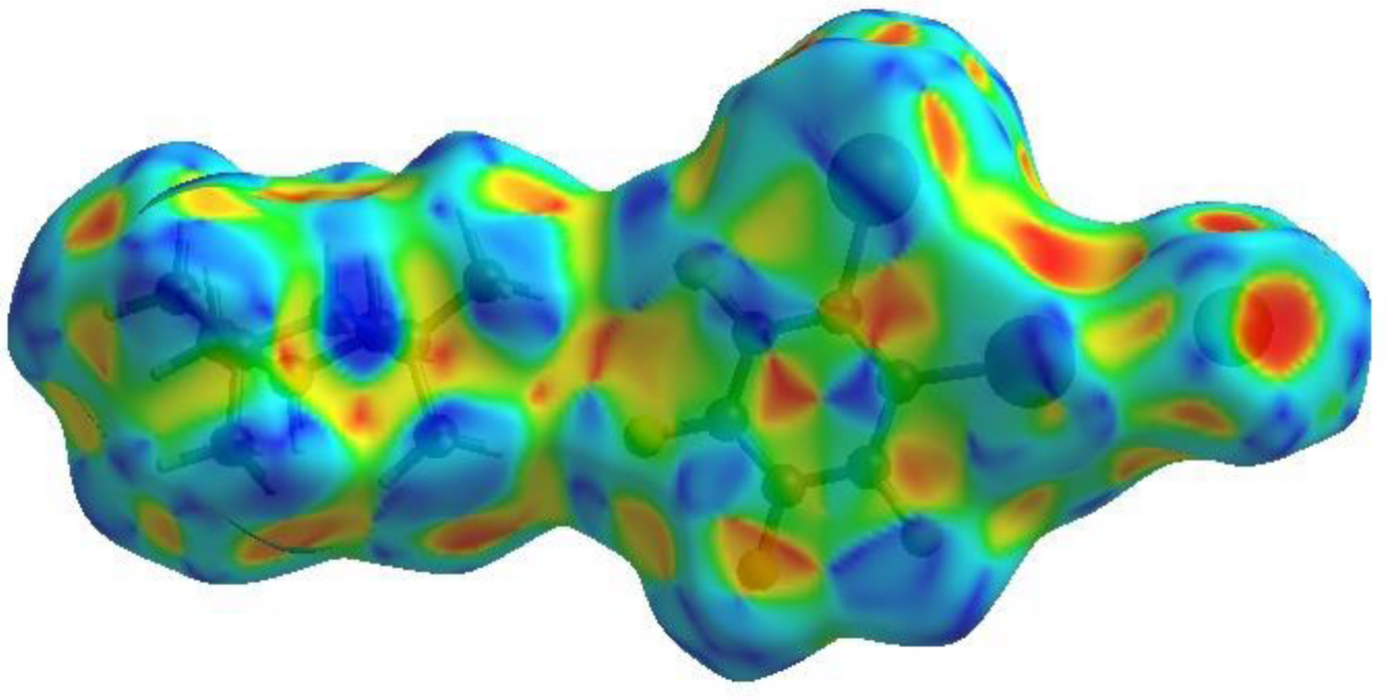
Hirshfeld surface of the title compound plotted over shape-index.

In order to visualize the inter­molecular inter­actions in the crystal of the title compound (I), a Hirshfeld surface (HS) analysis (Hirshfeld, 1977 ▸; Spackman & Jayatilaka, 2009 ▸) was carried out using *Crystal Explorer 17.5* (Spackman *et al.*, 2021 ▸). The contact distances *d*_i_ and *d*_e_ from the Hirshfeld surface to the nearest atom inside and outside, respectively, enable the analysis of the inter­molecular inter­actions through the mapping of *d*_norm_. The combination of *d*_i_ and *d*_e_ in the form of two-dimensional fingerprint plots (McKinnon *et al.*, 2004 ▸) provides a summary of inter­molecular contacts in the crystal. In the HS plotted over *d*_norm_ (Fig. 5 ▸), the white surface indicates contacts with distances equal to the sum of van der Waals radii, and the red and blue colours indicate distances shorter (in close contact) or longer (no/weak contact) than the van der Waals radii, respectively (Venkatesan *et al.*, 2016 ▸). The bright-red spots are indicative of their roles as the respective donors and/or acceptors. The shape-index would represent any C—H⋯π inter­action as red depressions located at the π-ring system and a blue region surrounding the respective C—H moiety, and hence Fig. 6 ▸ clearly suggests that there are no such C—H⋯π inter­actions present in (I). The shape-index of the HS can also indicate π–π stacking inter­actions by the presence of adjacent red and blue triangles. Fig. 6 ▸ suggests π–π inter­actions are present in (I).

**Figure 7:**
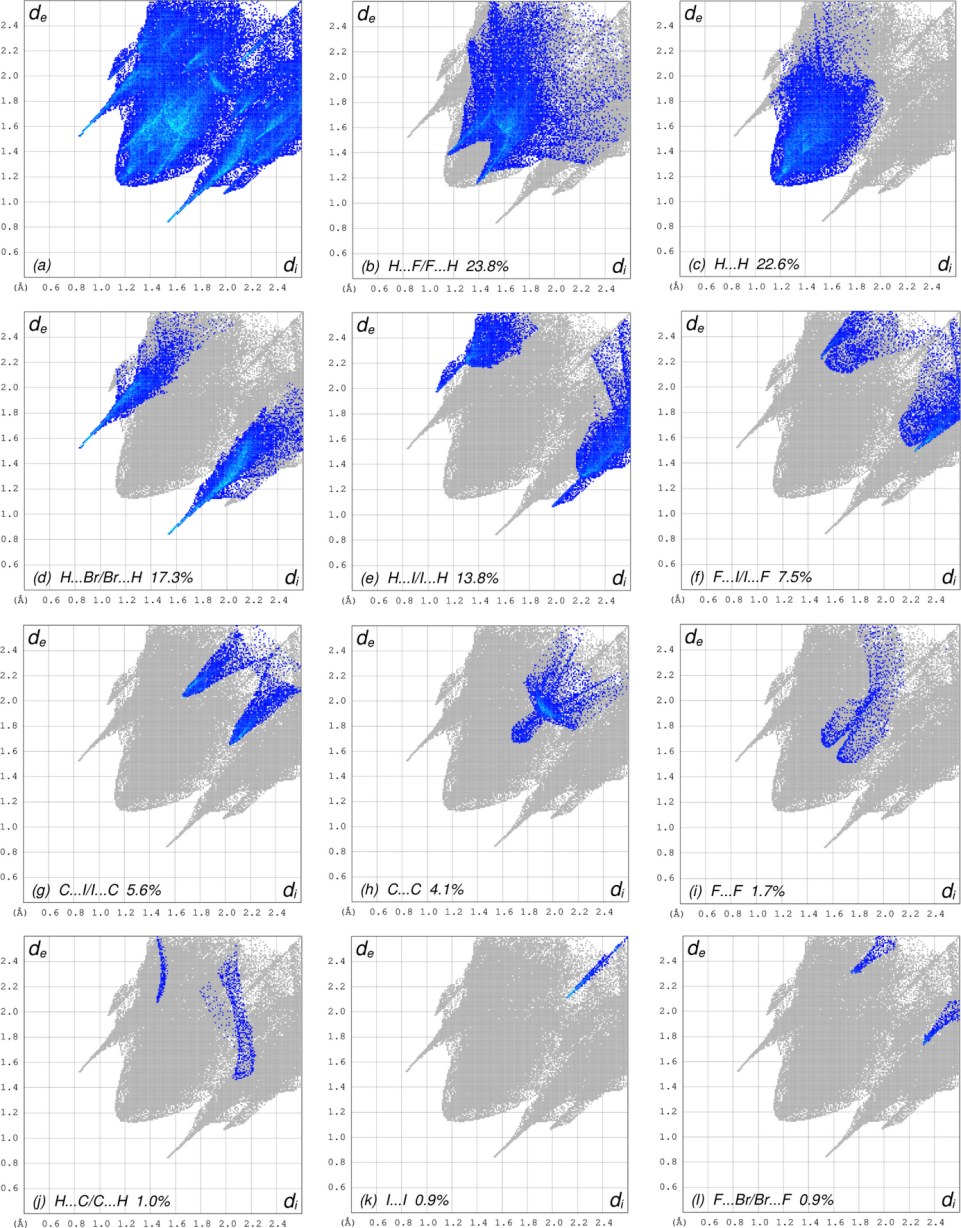
The full two-dimensional fingerprint plots for the title compound, showing (*a*) all inter­actions, and delineated into (*b*) H⋯F/F⋯H, (*c*) H⋯H, (*d*) H⋯Br/Br⋯H, (*e*) H⋯I/ ⋯H, (*f*) F⋯I/I⋯F, (*g*) C⋯I/I⋯C, (*h*) C⋯C, (*i*) F⋯F, (*j*) H⋯C/C⋯H, (*k*) I⋯I, and (*l*) F⋯Br/Br⋯F inter­actions. The *d*_i_ and *d*_e_ values are the closest inter­nal and external distances (in Å) from given points on the Hirshfeld surface.

The overall two-dimensional fingerprint plot is shown in Fig. 7 ▸*a* and those delineated into H⋯F/F⋯H, H⋯H, H⋯Br/Br⋯H, H⋯I/I⋯H, F⋯I/I⋯F, C⋯I/I⋯C, C⋯C, F⋯F, H⋯C/C⋯H, I⋯I and F⋯Br/Br⋯F contacts (McKinnon *et al.*, 2007 ▸) are illustrated in Fig. 7 ▸*b*–*l*, respectively, together with their relative contributions to the Hirshfeld surface. The most important inter­action clearly is of the H⋯F/F⋯H type (Table 2 ▸), contributing 23.8% to the overall crystal packing, which is reflected in Fig. 7 ▸*b* as pair of spikes with tips at *d*_e_ + *d*_i_ = 2.52 Å. The H⋯H inter­actions (Fig. 7 ▸*c*) contribute 22.6% to the HS and form a single maximum extension at *d*_e_ = *d*_i_ = 1.18 Å. The H⋯Br/Br⋯H (Fig. 7 ▸*d*) and H⋯I/I⋯H (Fig. 7 ▸*e*) contacts contribute 17.3% and 13.8%, respectively, to the HS, appearing as pairs of spikes with the tips at *d*_e_ + *d*_i_ = 2.36 Å and *d*_e_ + *d*_i_ = 3.04 Å, respectively. The F⋯I/I⋯F contacts (Fig. 7 ▸*f*) make a 7.5% contribution to the HS and have the tips at *d*_e_ + *d*_i_ = 3.74 Å. The C⋯I/I⋯C contacts (Fig. 7 ▸*g*) contribute 5.6%, the pair of spikes having tips at *d*_e_ + *d*_i_ = 3.70 Å. The C⋯C contacts (Fig. 7 ▸*h*) contribute 4.1% to the HS and have a bullet-shaped distribution of points with the tip at *d*_e_ = *d*_i_ = 1.68 Å. Finally, the F⋯F (Fig. 7 ▸*i*), H⋯C/C⋯H (Fig. 7 ▸*j*), I⋯I (Fig. 7 ▸*k*), F⋯Br/Br⋯F (Fig. 7 ▸*l*) and F⋯C/C⋯F (not shown) contacts make 1.7%, 1.0%, 0.9%, 0.9% and 0.8% contributions, respectively, to the HS and have very low densities of points.

**Figure 8:**
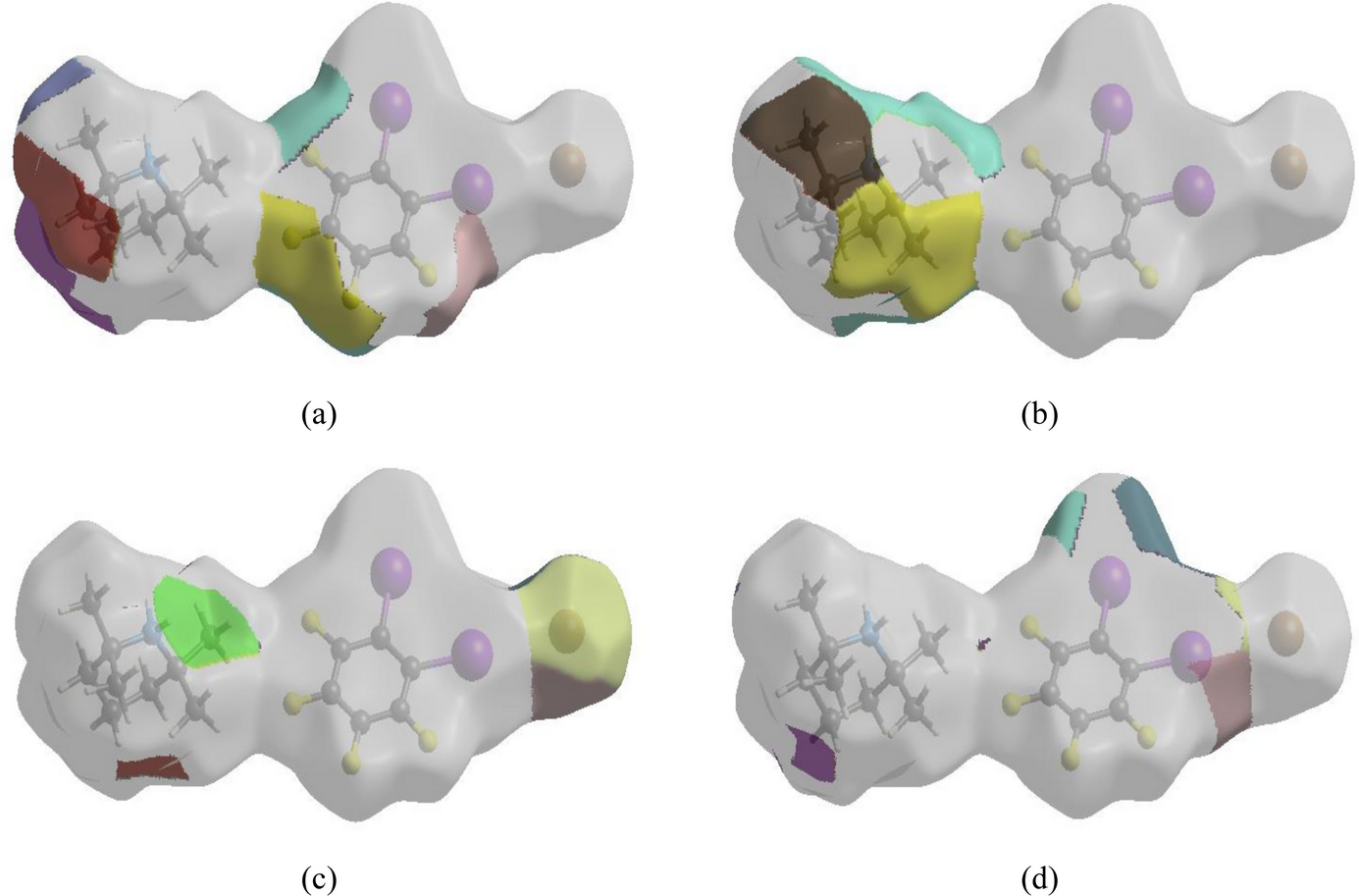
Hirshfeld surface representations of contact patches plotted onto the surface for (*a*) H⋯F/F⋯H, (*b*) H⋯H, (*c*) H⋯Br/Br⋯H and (*d*) H⋯I/I⋯H inter­actions.

The nearest neighbour coordination environment of a mol­ecule can be determined from the colour patches on the HS based on how close to other mol­ecules they are. The Hirshfeld surface representations with the fragment patches plotted onto the surface are shown for the H⋯F/F⋯H, H⋯H, H·· Br/Br⋯H and H⋯I/I⋯H inter­actions in Fig. 8 ▸*a*–*d*, respectively.

The Hirshfeld surface analysis confirms the importance of H-atom contacts in establishing the crystal packing. The large number of H⋯F/F⋯H, H⋯H, H⋯Br/Br⋯H and H⋯I/I⋯H inter­actions suggest that van der Waals inter­actions and hydrogen bonding play the major roles in the packing (Hathwar *et al.*, 2015 ▸).

## 5. Database survey

A survey of the Cambridge Structural Database (CSD, Version 5.42, last updated February 2023; Groom *et al.*, 2016 ▸) considering both ring motifs indicates that only one mol­ecular structure is closely related to the title compound (I), *viz*. 1-oxy-2,2,6,6-tetra­methyl­piperidin-4-yl radical benzoate bis­(1,2,3,4-tetra­fluoro-5, 6-di­iodo­benzene), C_16_H_22_NO_3_·2C_6_F_4_I_2_ (CSD refcode HISZEQ; Pang *et al.*, 2013 ▸). The C_6_F_4_I_2_ mol­ecules are essentially identical in their metrical parameters in both structures, while the aliphatic ring system in HISZEQ bears more substituents than in the title compound (I) resulting in small deviations of the overall geometries of the two respective six-membered rings.

## 6. Synthesis and crystallization

TEMPO (10 mmol), 1,2,3,4-tetra­fluoro-5,6-di­iodo­benzene (10 mmol) and CBr_4_ (10 mmol) were dissolved in 30 ml of hexa­ne/CH_2_Cl_2_ (*v*/*v*, 1:1), refluxed for 2 h, and left for slow evaporation. Orange crystals of the product started to form after 2 d at room temperature; they were filtered off and dried in air. Crystals suitable for X-ray analysis were obtained by slow evaporation of a methanol solution. Yield 61% (based on TEMPO), orange powder soluble in methanol, ethanol and DMSO. Analysis calculated for C_15_H_20_BrF_4_I_2_N (*M*_r_ = 624.04): C, 28.87; H, 3.23; N, 2.24. Found: C, 28.82; H, 3.20; N, 2.20. ^1^H NMR (DMSO-*d*^6^), δ: 8.08 (2N–H), 1.73 (2CH_2_), 1.65 (CH_2_), 1.31 (4CH_3_). ^13^C NMR (DMSO-*d*^6^), 15.6 (CH_2_), 26.9 (4CH_3_), 34.4 (2CH_2_), 57.2 [2C(CH_3_)_2_], 90.5 (2C—I), 148.1 (4C—F).

## 7. Refinement

Crystal data, data collection and structure refinement details are summarized in Table 3 ▸. The N-bound hydrogen atoms were located in a difference-Fourier map, and refined by applying restraints (DFIX). The C-bound H-atom positions were calculated geometrically at distances of 0.99 Å (for CH_2_) and 0.98 Å (for CH_3_) and refined using a riding model with *U*_iso_(H) = *k* × *U*_eq_(C), where *k* = 1.2 for CH_2_ hydrogen atoms and *k* = 1.5 for CH_3_ hydrogen atoms. Two reflections were omitted as clear outliers.

## Supplementary Material

Crystal structure: contains datablock(s) I. DOI: 10.1107/S2056989024011502/yz2062sup1.cif

Structure factors: contains datablock(s) I. DOI: 10.1107/S2056989024011502/yz2062Isup2.hkl

Supporting information file. DOI: 10.1107/S2056989024011502/yz2062Isup3.cml

CCDC reference: 2405482

Additional supporting information:  crystallographic information; 3D view; checkCIF report

## Figures and Tables

**Table 1:** Hydrogen-bond geometry (Å, °)

*D*—H⋯*A*	*D*—H	H⋯*A*	*D*⋯*A*	*D*—H⋯*A*
N1—H1*A*⋯Br1^ii^	0.86 (2)	2.55 (2)	3.407 (3)	179 (4)
N1—H1*B*⋯Br1^iii^	0.85 (2)	2.56 (2)	3.387 (3)	165 (5)
C13—H13*C*⋯Br1^ii^	0.98	2.91	3.767 (4)	147

Symmetry codes: (ii) 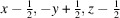; (iii) 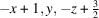.

**Table 2:** Selected interatomic distances (Å)

I1⋯F4	3.138 (3)	C11⋯H14*B*	2.83
I1⋯I2	3.7118 (4)	C12⋯H11*B*	2.83
I2⋯F1	3.111 (3)	C12⋯H14*B*	2.67
I1⋯H15*C*^i^	3.16	C14⋯H12*B*	2.72
H1*A*⋯Br1^ii^	2.546 (19)	C14⋯H11*B*	2.90
H13*C*⋯Br1^ii^	2.91	H1*A*⋯H13*C*	2.21
H1*B*⋯Br1^iii^	2.56 (2)	H1*A*⋯H15*C*	2.30
F1⋯F2	2.670 (4)	H1*B*⋯H12*C*	2.19
F2⋯F3	2.709 (4)	H1*B*⋯H14*A*	2.34
F3⋯F4	2.654 (4)	H7*B*⋯H15*C*	2.40
F2⋯H12*A*	2.65	H11*B*⋯H12*B*	2.19
H11*A*⋯F3^iv^	2.63	H11*B*⋯H14*B*	2.29
C12⋯C14	3.203 (6)	H12*B*⋯H14*B*	1.97
C11⋯H12*B*	2.76		

Symmetry codes: (i) 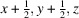; (ii) 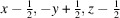; (iii) 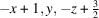; (iv) 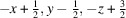.

**Table 3:** Experimental details

Crystal data	
Chemical formula	C_9_H_20_N^+^·Br^−^·C_6_F_4_I_2_
*M*_r_	624.03
Crystal system, space group	Monoclinic, *C*2/*c*
Temperature (K)	150
*a*, *b*, *c* (Å)	29.0324 (8), 9.1477 (2), 15.0296 (4)
β (°)	108.533 (1)
*V* (Å^3^)	3784.56 (17)
*Z*	8
Radiation type	Mo *K*α
μ (mm^−1^)	5.47
Crystal size (mm)	0.25 × 0.21 × 0.19

Data collection	
Diffractometer	Bruker APEXII CCD
Absorption correction	Multi-scan (*SADABS*; Krause *et al.*, 2015 ▸)
*T*_min_, *T*_max_	0.342, 0.423
No. of measured, independent and observed [*I* > 2σ(*I*)] reflections	14152, 3877, 3480
*R*_int_	0.022
(sin θ/λ)_max_ (Å^−1^)	0.627

Refinement	
*R*[*F*^2^ > 2σ(*F*^2^)], *wR*(*F*^2^), *S*	0.026, 0.069, 1.10
No. of reflections	3877
No. of parameters	220
No. of restraints	2
H-atom treatment	H atoms treated by a mixture of independent and constrained refinement
Δρ_max_, Δρ_min_ (e Å^−3^)	1.50, −0.69

Computer programs: *APEX4* and *SAINT* (Bruker, 2014 ▸), *SHELXT2019/1* (Sheldrick, 2015*a* ▸), *SHELXL2019/1* (Sheldrick, 2015*b* ▸) and *SHELXTL* (Sheldrick, 2008 ▸).

## supplementary crystallographic information

### (I) Crystal data

**Table:**

C_9_H_20_N^+^·Br^−^·C_6_F_4_I_2_	*F*(000) = 2352
*M**_r_* = 624.03	*D*_x_ = 2.190 Mg m^−^^3^
Monoclinic, *C*2/*c*	Mo *K*α radiation, λ = 0.71073 Å
*a* = 29.0324 (8) Å	Cell parameters from 8061 reflections
*b* = 9.1477 (2) Å	θ = 2.4–26.4°
*c* = 15.0296 (4) Å	µ = 5.47 mm^−^^1^
β = 108.533 (1)°	*T* = 150 K
*V* = 3784.56 (17) Å^3^	Prism, orange
*Z* = 8	0.25 × 0.21 × 0.19 mm

### (I) Data collection

**Table:**

Bruker APEXII CCD diffractometer	3480 reflections with *I* > 2σ(*I*)
φ and ω scans	*R*_int_ = 0.022
Absorption correction: multi-scan (SADABS; Krause *et al.*, 2015)	θ_max_ = 26.5°, θ_min_ = 2.6°
*T*_min_ = 0.342, *T*_max_ = 0.423	*h* = −36→33
14152 measured reflections	*k* = −11→11
3877 independent reflections	*l* = −18→18

### (I) Refinement

**Table:**

Refinement on *F*^2^	2 restraints
Least-squares matrix: full	Hydrogen site location: mixed
*R*[*F*^2^ > 2σ(*F*^2^)] = 0.026	H atoms treated by a mixture of independent and constrained refinement
*wR*(*F*^2^) = 0.069	*w* = 1/[σ^2^(*F*_o_^2^) + (0.0297*P*)^2^ + 23.4044*P*] where *P* = (*F*_o_^2^ + 2*F*_c_^2^)/3
*S* = 1.10	(Δ/σ)_max_ = 0.003
3877 reflections	Δρ_max_ = 1.50 e Å^−^^3^
220 parameters	Δρ_min_ = −0.69 e Å^−^^3^

### (I) Special details

**Table:**

Geometry. All esds (except the esd in the dihedral angle between two l.s. planes) are estimated using the full covariance matrix. The cell esds are taken into account individually in the estimation of esds in distances, angles and torsion angles; correlations between esds in cell parameters are only used when they are defined by crystal symmetry. An approximate (isotropic) treatment of cell esds is used for estimating esds involving l.s. planes.

### (I) Fractional atomic coordinates and isotropic or equivalent isotropic displacement parameters (Å^2^)

**Table:**

	*x*	*y*	*z*	*U*_iso_*/*U*_eq_	
I1	0.58724 (2)	0.50666 (3)	0.95416 (2)	0.02186 (8)	
I2	0.49598 (2)	0.20828 (3)	0.88878 (2)	0.03147 (9)	
Br1	0.70229 (2)	0.44644 (4)	1.06238 (3)	0.02025 (10)	
F1	0.39660 (9)	0.3559 (3)	0.7874 (2)	0.0358 (6)	
F2	0.36882 (9)	0.6336 (3)	0.7483 (2)	0.0385 (7)	
F3	0.43515 (10)	0.8520 (3)	0.7953 (2)	0.0402 (7)	
F4	0.52828 (10)	0.7943 (3)	0.8811 (2)	0.0350 (6)	
N1	0.20902 (12)	0.4228 (4)	0.5387 (2)	0.0168 (7)	
C1	0.41559 (15)	0.6043 (5)	0.7915 (3)	0.0273 (10)	
C2	0.43063 (15)	0.4616 (5)	0.8126 (3)	0.0251 (9)	
C3	0.47865 (15)	0.4272 (5)	0.8587 (3)	0.0223 (8)	
C4	0.51296 (14)	0.5399 (5)	0.8839 (3)	0.0208 (8)	
C5	0.49698 (15)	0.6813 (5)	0.8607 (3)	0.0247 (9)	
C6	0.44896 (16)	0.7134 (5)	0.8155 (3)	0.0285 (10)	
C7	0.12586 (15)	0.4790 (5)	0.5367 (3)	0.0258 (9)	
H7A	0.095235	0.529016	0.501617	0.031*	
H7B	0.117866	0.376129	0.546160	0.031*	
C8	0.16011 (14)	0.4814 (4)	0.4774 (3)	0.0203 (8)	
C9	0.23227 (14)	0.4809 (4)	0.6381 (3)	0.0200 (8)	
C10	0.19400 (15)	0.4749 (5)	0.6869 (3)	0.0241 (9)	
H10A	0.207115	0.520952	0.749488	0.029*	
H10B	0.186794	0.371383	0.696339	0.029*	
C11	0.14695 (15)	0.5518 (5)	0.6321 (3)	0.0270 (9)	
H11A	0.123373	0.545906	0.667202	0.032*	
H11B	0.153481	0.656302	0.623706	0.032*	
C12	0.25235 (15)	0.6339 (5)	0.6354 (3)	0.0261 (9)	
H12A	0.273188	0.661239	0.698410	0.039*	
H12B	0.225401	0.703502	0.614153	0.039*	
H12C	0.271380	0.635554	0.592027	0.039*	
C13	0.27448 (14)	0.3797 (5)	0.6841 (3)	0.0249 (9)	
H13A	0.289234	0.407421	0.750087	0.037*	
H13B	0.298770	0.387430	0.651613	0.037*	
H13C	0.262726	0.278711	0.680484	0.037*	
C14	0.16547 (16)	0.6325 (5)	0.4403 (3)	0.0290 (10)	
H14A	0.191404	0.631076	0.411518	0.043*	
H14B	0.173643	0.702913	0.492089	0.043*	
H14C	0.134833	0.661096	0.393177	0.043*	
C15	0.14272 (15)	0.3773 (5)	0.3950 (3)	0.0245 (9)	
H15A	0.165817	0.377765	0.359601	0.037*	
H15B	0.110710	0.408459	0.354083	0.037*	
H15C	0.140398	0.278296	0.418190	0.037*	
H1A	0.2074 (14)	0.330 (2)	0.544 (3)	0.010 (10)*	
H1B	0.2309 (15)	0.451 (6)	0.517 (4)	0.040 (15)*	

### (I) Atomic displacement parameters (Å^2^)

**Table:**

	*U*^11^	*U*^22^	*U*^33^	*U*^12^	*U*^13^	*U*^23^
I1	0.01724 (13)	0.02663 (16)	0.02143 (14)	−0.00053 (10)	0.00575 (10)	−0.00051 (11)
I2	0.03171 (16)	0.02433 (16)	0.03802 (18)	−0.00134 (12)	0.01060 (13)	0.00187 (12)
Br1	0.01935 (19)	0.0180 (2)	0.0230 (2)	0.00066 (15)	0.00620 (16)	−0.00154 (16)
F1	0.0249 (13)	0.0378 (16)	0.0414 (16)	−0.0114 (12)	0.0058 (12)	0.0024 (13)
F2	0.0188 (12)	0.0524 (18)	0.0406 (16)	0.0086 (12)	0.0040 (11)	0.0072 (14)
F3	0.0398 (15)	0.0286 (15)	0.0507 (18)	0.0130 (12)	0.0121 (14)	0.0063 (14)
F4	0.0361 (14)	0.0235 (14)	0.0428 (16)	−0.0082 (11)	0.0091 (12)	−0.0004 (12)
N1	0.0200 (17)	0.0119 (17)	0.0199 (17)	−0.0013 (13)	0.0083 (14)	0.0011 (13)
C1	0.020 (2)	0.041 (3)	0.022 (2)	0.0036 (18)	0.0077 (17)	0.0035 (19)
C2	0.020 (2)	0.035 (2)	0.021 (2)	−0.0069 (18)	0.0077 (17)	0.0000 (18)
C3	0.025 (2)	0.023 (2)	0.019 (2)	0.0004 (17)	0.0087 (17)	0.0012 (17)
C4	0.0174 (19)	0.026 (2)	0.020 (2)	−0.0006 (16)	0.0067 (16)	−0.0018 (17)
C5	0.025 (2)	0.029 (2)	0.022 (2)	−0.0012 (18)	0.0087 (17)	−0.0022 (18)
C6	0.029 (2)	0.032 (3)	0.026 (2)	0.0066 (19)	0.0115 (19)	0.0037 (19)
C7	0.020 (2)	0.026 (2)	0.030 (2)	0.0017 (17)	0.0072 (18)	0.0010 (19)
C8	0.0186 (19)	0.017 (2)	0.025 (2)	0.0028 (15)	0.0069 (16)	0.0021 (17)
C9	0.0211 (19)	0.020 (2)	0.0185 (19)	−0.0034 (16)	0.0057 (16)	−0.0031 (16)
C10	0.026 (2)	0.026 (2)	0.024 (2)	−0.0024 (17)	0.0127 (18)	−0.0027 (18)
C11	0.025 (2)	0.029 (2)	0.030 (2)	0.0013 (18)	0.0128 (18)	−0.0042 (19)
C12	0.022 (2)	0.024 (2)	0.034 (2)	−0.0055 (17)	0.0112 (18)	−0.0086 (19)
C13	0.021 (2)	0.029 (2)	0.023 (2)	0.0007 (17)	0.0048 (17)	−0.0022 (18)
C14	0.031 (2)	0.020 (2)	0.034 (2)	0.0040 (18)	0.0088 (19)	0.0077 (19)
C15	0.024 (2)	0.023 (2)	0.023 (2)	−0.0002 (17)	0.0030 (17)	0.0022 (17)

### (I) Geometric parameters (Å, º)

**Table:**

I1—C4	2.100 (4)	C8—C14	1.517 (6)
I2—C3	2.080 (4)	C9—C10	1.514 (5)
F1—C2	1.348 (5)	C9—C13	1.516 (6)
F2—C1	1.333 (5)	C9—C12	1.522 (6)
F3—C6	1.335 (5)	C10—C11	1.525 (6)
F4—C5	1.346 (5)	C10—H10A	0.9900
N1—C8	1.523 (5)	C10—H10B	0.9900
N1—C9	1.526 (5)	C11—H11A	0.9900
N1—H1A	0.860 (19)	C11—H11B	0.9900
N1—H1B	0.846 (19)	C12—H12A	0.9800
C1—C6	1.357 (7)	C12—H12B	0.9800
C1—C2	1.381 (7)	C12—H12C	0.9800
C2—C3	1.382 (6)	C13—H13A	0.9800
C3—C4	1.399 (6)	C13—H13B	0.9800
C4—C5	1.381 (6)	C13—H13C	0.9800
C5—C6	1.375 (6)	C14—H14A	0.9800
C7—C11	1.522 (6)	C14—H14B	0.9800
C7—C8	1.531 (6)	C14—H14C	0.9800
C7—H7A	0.9900	C15—H15A	0.9800
C7—H7B	0.9900	C15—H15B	0.9800
C8—C15	1.516 (6)	C15—H15C	0.9800
			
I1···F4	3.138 (3)	C11···H14B	2.83
I1···I2	3.7118 (4)	C12···H11B	2.83
I2···F1	3.111 (3)	C12···H14B	2.67
H1A···Br1^i^	2.56	C14···H12B	2.72
H13C···Br1^i^	2.91	C14···H11B	2.90
H1B···Br1^ii^	2.58 (5)	H1A···H13C	2.22
F1···F2	2.670 (4)	H1A···H15C	2.30
F2···F3	2.709 (4)	H1B···H12C	2.16
F3···F4	2.654 (4)	H1B···H14A	2.32
F2···H12A	2.65	H7B···H15C	2.40
H11A···F3^iii^	2.63	H11B···H12B	2.19
C12···C14	3.203 (6)	H11B···H14B	2.27
C11···H12B	2.76	H12B···H14B	1.97
			
C8—N1—C9	120.4 (3)	C10—C9—N1	107.3 (3)
C8—N1—H1A	110 (3)	C13—C9—N1	106.0 (3)
C9—N1—H1A	106 (3)	C12—C9—N1	110.4 (3)
C8—N1—H1B	109 (4)	C9—C10—C11	113.0 (4)
C9—N1—H1B	97 (4)	C9—C10—H10A	109.0
H1A—N1—H1B	114 (5)	C11—C10—H10A	109.0
F2—C1—C6	120.8 (4)	C9—C10—H10B	109.0
F2—C1—C2	120.1 (4)	C11—C10—H10B	109.0
C6—C1—C2	119.2 (4)	H10A—C10—H10B	107.8
F1—C2—C1	117.6 (4)	C7—C11—C10	109.3 (3)
F1—C2—C3	120.7 (4)	C7—C11—H11A	109.8
C1—C2—C3	121.7 (4)	C10—C11—H11A	109.8
C2—C3—C4	119.1 (4)	C7—C11—H11B	109.8
C2—C3—I2	117.7 (3)	C10—C11—H11B	109.8
C4—C3—I2	123.3 (3)	H11A—C11—H11B	108.3
C5—C4—C3	117.9 (4)	C9—C12—H12A	109.5
C5—C4—I1	118.1 (3)	C9—C12—H12B	109.5
C3—C4—I1	124.0 (3)	H12A—C12—H12B	109.5
F4—C5—C6	117.0 (4)	C9—C12—H12C	109.5
F4—C5—C4	120.9 (4)	H12A—C12—H12C	109.5
C6—C5—C4	122.1 (4)	H12B—C12—H12C	109.5
F3—C6—C1	120.0 (4)	C9—C13—H13A	109.5
F3—C6—C5	119.9 (4)	C9—C13—H13B	109.5
C1—C6—C5	120.0 (4)	H13A—C13—H13B	109.5
C11—C7—C8	113.6 (4)	C9—C13—H13C	109.5
C11—C7—H7A	108.8	H13A—C13—H13C	109.5
C8—C7—H7A	108.8	H13B—C13—H13C	109.5
C11—C7—H7B	108.8	C8—C14—H14A	109.5
C8—C7—H7B	108.8	C8—C14—H14B	109.5
H7A—C7—H7B	107.7	H14A—C14—H14B	109.5
C15—C8—C14	108.6 (4)	C8—C14—H14C	109.5
C15—C8—N1	106.1 (3)	H14A—C14—H14C	109.5
C14—C8—N1	111.0 (3)	H14B—C14—H14C	109.5
C15—C8—C7	110.9 (3)	C8—C15—H15A	109.5
C14—C8—C7	112.9 (3)	C8—C15—H15B	109.5
N1—C8—C7	107.2 (3)	H15A—C15—H15B	109.5
C10—C9—C13	111.6 (3)	C8—C15—H15C	109.5
C10—C9—C12	113.1 (3)	H15A—C15—H15C	109.5
C13—C9—C12	108.2 (3)	H15B—C15—H15C	109.5
			
F2—C1—C2—F1	0.4 (6)	C2—C1—C6—C5	−0.4 (6)
C6—C1—C2—F1	−179.7 (4)	F4—C5—C6—F3	−1.2 (6)
F2—C1—C2—C3	−178.9 (4)	C4—C5—C6—F3	179.3 (4)
C6—C1—C2—C3	1.0 (6)	F4—C5—C6—C1	179.0 (4)
F1—C2—C3—C4	−179.9 (4)	C4—C5—C6—C1	−0.6 (7)
C1—C2—C3—C4	−0.6 (6)	C9—N1—C8—C15	−166.9 (3)
F1—C2—C3—I2	−0.7 (5)	C9—N1—C8—C14	75.4 (4)
C1—C2—C3—I2	178.6 (3)	C9—N1—C8—C7	−48.4 (4)
C2—C3—C4—C5	−0.4 (6)	C11—C7—C8—C15	166.3 (4)
I2—C3—C4—C5	−179.5 (3)	C11—C7—C8—C14	−71.7 (5)
C2—C3—C4—I1	180.0 (3)	C11—C7—C8—N1	50.9 (5)
I2—C3—C4—I1	0.9 (5)	C8—N1—C9—C10	49.8 (4)
C3—C4—C5—F4	−178.6 (4)	C8—N1—C9—C13	169.1 (3)
I1—C4—C5—F4	1.1 (5)	C8—N1—C9—C12	−73.8 (4)
C3—C4—C5—C6	1.0 (6)	C13—C9—C10—C11	−169.0 (4)
I1—C4—C5—C6	−179.4 (3)	C12—C9—C10—C11	68.7 (5)
F2—C1—C6—F3	−0.4 (6)	N1—C9—C10—C11	−53.2 (4)
C2—C1—C6—F3	179.7 (4)	C8—C7—C11—C10	−58.9 (5)
F2—C1—C6—C5	179.5 (4)	C9—C10—C11—C7	60.2 (5)

Symmetry codes: (i) *x*−1/2, −*y*+1/2, *z*−1/2; (ii) −*x*+1, *y*, −*z*+3/2; (iii) −*x*+1/2, *y*−1/2, −*z*+3/2.

### (I) Hydrogen-bond geometry (Å, º)

**Table:**

*D*—H···*A*	*D*—H	H···*A*	*D*···*A*	*D*—H···*A*
N1—H1*A*···Br1^i^	0.86 (2)	2.55 (2)	3.409 (3)	179 (4)
N1—H1*B*···Br1^ii^	0.85 (2)	2.58 (3)	3.387 (3)	161 (5)
C13—H13*C*···Br1^i^	0.98	2.91	3.769 (4)	146

Symmetry codes: (i) *x*−1/2, −*y*+1/2, *z*−1/2; (ii) −*x*+1, *y*, −*z*+3/2.
